# Evaluation of parasite subpopulations and genetic diversity of the *msp1, msp2* and *glurp* genes during and following artesunate monotherapy treatment of *Plasmodium falciparum* malaria in Western Cambodia

**DOI:** 10.1186/1475-2875-12-403

**Published:** 2013-11-09

**Authors:** Panita Gosi, Charlotte A Lanteri, Stuart D Tyner, Youry Se, Chanthap Lon, Michele Spring, Mengchuor Char, Darapiseth Sea, Sabaithip Sriwichai, Sittidech Surasri, Saowaluk Wongarunkochakorn, Kingkan Pidtana, Douglas S Walsh, Mark M Fukuda, Jessica Manning, David L Saunders, Delia Bethell

**Affiliations:** 1 Department of Immunology and Medicine, Armed Forces Research Institute of Medical Science (AFRIMS), Bangkok, Thailand; 2 Armed Forces Research Institute of Medical Science (AFRIMS), Phnom Penh, Cambodia; 3 National Center for Parasitology, Entomology and Malaria Control, Phnom Penh, Cambodia

**Keywords:** Malaria, *Plasmodium falciparum*, Cambodia, Genotype, Artesunate, *msp1*, *msp2*, *glurp*

## Abstract

**Background:**

Despite widespread coverage of the emergence of artemisinin resistance, relatively little is known about the parasite populations responsible. The use of PCR genotyping around the highly polymorphic *Plasmodium falciparum msp1*, *msp2* and *glurp* genes has become well established both to describe variability in alleles within a population of parasites, as well as classify treatment outcome in cases of recurrent disease. The primary objective was to assess the emergence of minority parasite clones during seven days of artesunate (AS) treatment in a location with established artemisinin resistance. An additional objective was to investigate whether the classification of clinical outcomes remained valid when additional genotyping was performed.

**Methods:**

Blood for parasite genotyping was collected from 143 adult patients presenting with uncomplicated falciparum malaria during a clinical trial of AS monotherapy in Western Cambodia. Nested allelic type-specific amplification of the genes encoding the merozoite surface proteins 1 and 2 (*msp1* and *msp2*) and the glutamate-rich protein (*glurp*) was performed at baseline, daily during seven days of treatment, and again at failure. Allelic variants were analysed with respect to the size of polymorphisms using Quantity One software to enable identification of polyclonal infections.

**Results:**

Considerable variation of *msp2* alleles but well-conserved *msp1* and *glurp* were identified. At baseline, 31% of infections were polyclonal for one or more genes. Patients with recurrent malaria were significantly more likely to have polyclonal infections than patients without recurrence (seven of nine *versus* 36 of 127, p = 0.004). Emergence of minority alleles during treatment was detected in only one of twenty-three cases defined as being artemisinin resistant. Moreover, daily genotyping did not alter the final outcome classification in any recurrent cases.

**Conclusions:**

The parasites responsible for artemisinin-resistant malaria in a clinical trial in Western Cambodia comprise the dominant clones of acute malaria infections rather than minority clones emerging during treatment. Additional genotyping during therapy was not beneficial. Disproportionately high rates of polyclonal infections in cases of recurrence suggest complex infections lead to poor treatment outcomes. Current research objectives should be broadened to include identification and follow-up of recurrent polyclonal infections so as to define their role as potential agents of emerging resistance.

## Background

With the emergence of artemisinin resistance in Western Cambodia and along its international border with Thailand as well as further afield in Vietnam and along Thailand’s western border with Myanmar, there is a public health mandate to characterize the parasite populations responsible for poor clinical outcomes, as well as better understand the dynamics of how these parasite clones co-exist [1-3]. Gene flow studies and complex whole genome sequencing analyses offer highly sophisticated approaches and have already been employed in this region in an attempt to define the aetiology of the emergence and spread of resistance [4,5]. However these approaches are costly and require considerable expertise both to analyse samples and interpret the data.

The use of polymerase chain reaction (PCR) genotyping around a limited number of polymorphic genes, which are known to be highly variable in *Plasmodium falciparum* parasites, has become well established both to describe variability in alleles within a population of parasites, as well as to distinguish recrudescence from new infection in cases of recurrent disease [6-8]. This technique is relatively cost-efficient and straightforward to perform without requiring extensive sequencing and bioinformatics capabilities. The technique provides a basic description of parasite populations and diversity within a patient cohort using these highly variable parasite genes, as well as classification of recurrent malaria episodes as recrudescent (true failures) or new infections. The most widely used PCR genotyping assay involves nested allelic type-specific amplification of the genes encoding the merozoite surface proteins 1 and 2 (*msp1* and *msp2*) and the glutamate-rich protein (*glurp*) [8,9]. This method is highly sensitive and allows for classification of allelic variants including minor variants in mixed clonal infections [10]. The use of high-resolution methods, such as repeated agarose gel electrophoresis with computer image software, for determining DNA band size has increased the ability to distinguish and analyse PCR products that differ only slightly in size and thus have improved the accuracy of this technique. MSP1 block 2 allelic variants fall under three major types - MAD20, K1 and RO33. The *msp2* gene can be similarly grouped into two allelic families, FC27 and IC3D7 [11,12]. Only size variation, due to the different number of repeats, has been observed within the *glurp* gene [9].

The present analysis was conducted as part of a clinical trial of artesunate (AS) monotherapy designed to probe for and characterize clinical artemisinin resistance in adult patients with uncomplicated *P. falciparum* malaria. The trial was conducted in a rural district of Western Cambodia, close to the international border with Thailand and where reports of AS treatment failures had already emerged [13]. The main objective of this study was to describe changes in parasite genotype during seven days of AS treatment in patients classified as artemisinin resistant and assess for emergence of novel (minority) parasite populations that were not detected at baseline. Secondary objectives were: (1) to describe the diversity of *msp1*, *msp2* and *glurp* in this Cambodian study population, and (2) to assess whether conventional classification of recrudescence/re-infection based on samples collected only at baseline and day of failure (D_f_) remained valid once interim sampling was performed. There were two working hypotheses. Firstly, that AS-resistant patients were infected with novel subpopulations of relatively drug-resistant parasites not identified at baseline; as the majority of more sensitive parasites were killed, the resistant parasites would persist and become detectable during a seven-day course of AS therapy. Secondly, that new falciparum infections during follow-up had persistence of these intrinsically more resistant parasite subpopulations. Hence, these cases should be reclassified as recrudescent, making the true failure rate higher with longitudinal genotyping compared to the conventional time points of baseline and D_f_.

## Methods

### Clinical methods

#### Study setting and participants

The study was part of a randomized clinical trial conducted during 2008-2009 at Tasanh Health Center located in Battambang Province in Western Cambodia, a region of low malaria transmission, with just one third of the study population reporting one or more previous episodes of malaria. Full details of the study are described elsewhere [14]. Briefly, after giving informed consent, screening procedures were conducted, and eligible patients with uncomplicated *P. falciparum* malaria were randomized to receive one of three AS monotherapy regimens given as a single daily oral dose: 2, 4 or 6 mg/kg/day (n = 75, 40, and 28, respectively) for seven days. Inclusion criteria were age 18 to 65 years, fever or history of fever within 48 hours of presentation, mono-infection with *P. falciparum* as determined on Giemsa-stained thick and thin blood films by light microscopy, and parasite density 1,000-200,000 asexual parasites/ml blood. Exclusion criteria were signs and symptoms of severe malaria and documented use of anti-malarial drugs within the preceding 30 days. All patients remained on the study ward for the first week until completion of AS administration and returned for weekly outpatient review until Day 42. Patient outcomes for the main treatment study were classified according to clinical and parasitological responses [15]. Primary analysis for this molecular study was intent-to-treat so as to include additional subjects who had given informed consent to participate, including withdrawal and screening failure cases where a baseline blood sample for molecular genotyping had been collected. The study was approved by the WRAIR IRB, World Health Organization Ethical Review Committee (WHO ERC), and the Cambodian Ethics Committee for Human Research (NECHR).

For the longitudinal genotyping analysis conducted over the first seven days (Days 0 to 6), three subgroups of patients were selected and defined as follows: (Group A) “artemisinin sensitive” (rapid resolution of symptoms plus asexual parasite clearance time <48 hours by microscopy and remained cured to 42 days) (n = 18); (Group B) “slow parasite clearance” (parasite clearance time >96 hours by microscopy and remained cured of *P. falciparum* throughout the follow up period (n = 14); (Group C) “Recurrence” (re-appearance of asexual *P. falciparum* parasites, as determined by light microscopy, during follow up after initially clearing parasitaemia, with or without clinical symptoms/signs of malaria (n = 9). Groups B and C when combined were termed “artemisinin resistant”. Group D constituted the remaining patients not included in A, B or C. For the purposes of the present analysis, enrolled patients developing *Plasmodium vivax* mono-infection during follow-up and confirmed by vivax-specific PCR were considered cured of *P. falciparum* infection.

#### Sample collection

Prior to initiation of AS therapy (Day 0), blood for parasite density, species confirmation and genotyping was collected into EDTA. Further samples were collected daily on Days 1 to 6 and again on the day of treatment failure (D_f_) if malaria recurrence occurred. All samples were transferred to cryovials, stored in liquid nitrogen at the study site for transfer to Bangkok, and then kept frozen at -80°C until used for DNA extraction.

### Laboratory methods

#### Genotyping of *Plasmodium falciparum*

Genomic DNA was extracted from 200 μl whole blood per patient using the Qiagen DNA extraction kit (QIAGEN, USA) according to manufacturer’s instructions. The polymorphic regions of the merozoite surface proteins *msp1* (block 2), *msp2* (block 3), and *glurp* (R2 repeat region) were amplified by nested PCR. In the primary reaction, the oligonucleotide primers corresponded to conserved sequences within *msp1* (block 2), *msp2* (block 3) and *glurp*, and in the nested reaction, separate primer pairs targeted the respective allelic types of *msp1* (K1, MAD20, and RO33) and *msp2* (FC27 and IC3D7) for amplification [11]. For controls, genomic DNA from a 3D7 laboratory strain was isolated by standard techniques. Positive and negative controls were systematically incorporated in each PCR run. The *msp1, msp2* and *glurp* PCR products were loaded on 2% agarose gels, stained with ethidium bromide, separated by electrophoresis and visualized under UV trans-illumination (VersaDoc®, BIORAD, Hercules, USA). Samples from an individual patient were run in adjacent lanes. If there was no amplification for any allelic family, the PCR was repeated with three times the quantity of template DNA. If no amplification was detected after this second reaction, amplification was classified as unsuccessful. Analyses of number of genotypes and size polymorphism were digitalized using Quantity One® software (Bio-Rad Laboratories, Hercules, USA). A minimum of ten base pairs size difference was required to define an additional genotype.

#### Determination of PCR-corrected response

Comparison of genotyping patterns of *msp1*, *msp2* and *glurp* at baseline and at D_f_ was used to classify parasitological outcomes for LTF cases. Genotyping was done stepwise with an initial *msp2* determination, followed by *msp1* genotyping of the paired blood samples found to have at least one identical *msp2* allele before and after treatment. Based on analysis of the 3D7 strain, alleles were considered the same if molecular weights were within ten base pairs (bp) [2,6]. It was assumed that after a patient was initially treated for malaria, a subsequent malaria episode was caused by either parasite strains present before treatment (e.g., recrudescence) or parasite strains acquired after completion of treatment (e.g., new infection). An outcome was defined as recrudescence if a subsequent sample contained identical alleles or a subset of the alleles present in the first sample [8]. If a subsequent sample contained alleles present in the first sample and new alleles, the outcome was still considered recrudescent. An outcome was defined as a new infection if a subsequent sample contained only new alleles.

Allelic variants were analysed only with respect to the size of polymorphisms given the high level of discrimination afforded by the Quantity One® software computer analysis.

### Statistical methods

Continuous data were expressed as medians with interquartile ranges or, in the case of parasite counts, as geometric means with 95% confidence intervals (95% CI). Continuous data from patient groups were compared using the non-parametric Kruskall-Wallis test. Numerical data were expressed as proportions and compared using Chi-square or Fisher’s exact tests as appropriate. All statistical tests were performed at the 5% significance level and corresponding 95% confidence intervals were estimated. Statistical analyses were performed using SPSS version 12.0 (SPSS Inc., Chicago, IL, USA) and Stata version 11 (College Station, TX, USA).

## Results

PCR genotyping was successful for one or more genes in baseline samples from 143 patients who had undergone screening procedures prior to study enrolment (*msp1* 125, *msp2* 137 and *glurp* 143). Four patients were subsequently determined not to meet all treatment study entry criteria, but genotype samples from these cases were retained for this molecular analysis. Some 139 patients were randomized to one of three oral AS monotherapy regimens; three were subsequently lost to follow-up while the remaining 136 were followed until a study endpoint was met. One case re-presented with recurrence 56 days after enrolment into the treatment study and was included in this analysis; another developed neutropenia on D3 and was withdrawn from the treatment study but retained for safety follow up and developed malaria recurrence at D42. Baseline characteristics and clinical/parasitological outcomes for patients are given in Table 1. Subjects termed artemisinin-resistant (Groups B and C combined) had significantly higher presenting parasitaemia and slower parasite clearance rates and times than those in Group A (artemisinin-sensitive) (p < 0.0001 for all comparisons). Approximately one quarter of the study population, including nearly half of the malaria recurrence cases, had gametocytes documented by microscopy at some point during the 42-day study follow-up period; however, gametocytes were detected in only two cases at the same time a PCR genotyping sample was collected: one in Group A (at baseline) and one in Group C (at D_f_).

**Table 1:** **Baseline characteristics and clinical and parasitological outcomes in groups of patients with *Plasmodium falciparum *** **malaria followed with sequential parasite genotyping during 7 days of artesunate monotherapy**

	**Group A**	**Group B**	**Group C**	**Groups B + C**	**Group D**	**Overall study population**
	**Artemisinin “sensitive”**	**Slow parasite clearance**	**Recurrence**	**Artemisinin “resistant”**	**Remaining study cases**	
Number of cases	18	14	9	23	102	143
Age (y), median (IQR)	25 (18-32)	25 (21-31)	22 (18-27)	23 (20-31)	25 (20-38)	25 (20-35)
Male:female, n (%)	16:2 (89:11)	13:1 (93:7)	6:3 (67:33)	19:4 (83:17)	77:25 (75:25)	112:31 (78:22)
Parasitaemia (/uL), geometric mean (95%CI)	6,698 (3,564-12,589)	51,944 (28,540-94,542)	22,209 (9,193-53,653)	37,251 (22,776-60,926)	11,527 (4,933-32,687)	14,207 (4,891-37,778)
Allocated daily AS monotherapy dose: 2 - 4 - 6 (mg/kg/day), n^+^	10 - 5 - 3	3 - 8 - 3	4 - 2 - 3	7 - 10 - 6	58 - 25 - 19	71 - 40 - 28
Parasite clearance time by microscopy (h), median (IQR)	36 (30-42)	108 (102-108)	96 (84-96)	102 (96-108)	74 (66-84)	78 (60-90)
Parasite clearance rate* (/h), median (IQR)	0.086 (0.059-0.11)	0.036 (0.033-0.038)	0.035 (0.030-0.041)	0.036 (0.033-0.039)	0.040 (0.034-0.047)	0.040 (0.034-0.052)
Parasitological outcome at D42, n (%)						
Cured	18 (100)	14 (100)	1 (11)^	15 (65)^	96 (96)	131 (92)
Recrudescence**	0	0	7 (67)	7 (30)	0 (0)	7 (5)
New Pf infection**	0	0	1 (11)	1 (4)	0 (0)	1(1)
No outcome recorded	0	0	0	0	4 (4)^+^	4 (3)
Gametocytes by microscopy, n (%)						
At baseline	1 (6)	1 (7)	1 (11)	2 (9)	16 (16)	19 (13)
During week 1	3 (17)	1 (7)	1 (11)	1 (4)	26 (25)	30 (21)
Any time D0-42	3 (17)	0	4 (44)	4 (17)	29 (28)	36 (26)

Key. * Calculated using slope of curve for log_10_-normalised parasite clearance as determined by light microscopy [14].

^+^ 4 screening failure cases received standard AS-MQ treatment and had baseline data recorded only.

** Outcome determined using genotyping based on *msp1, msp2* and *glurp* fragment sizes on paired blood samples collected at baseline and D_f_.

^ Includes one case classified *per protocol* as cured on D42 but self-presented with recurrent malaria on D58 which was subsequently determined to be recrudescence by PCR genotyping.

h = hours; y = years.

Significant differences exist between artemisinin sensitive and resistant groups for parasitemia, parasite clearance time and parasite clearance rate (p = 0.0001 for all comparisons).

### Population diversity of *msp1*, *msp2* and *glurp* at baseline

#### Population diversity

**Figure 1:**
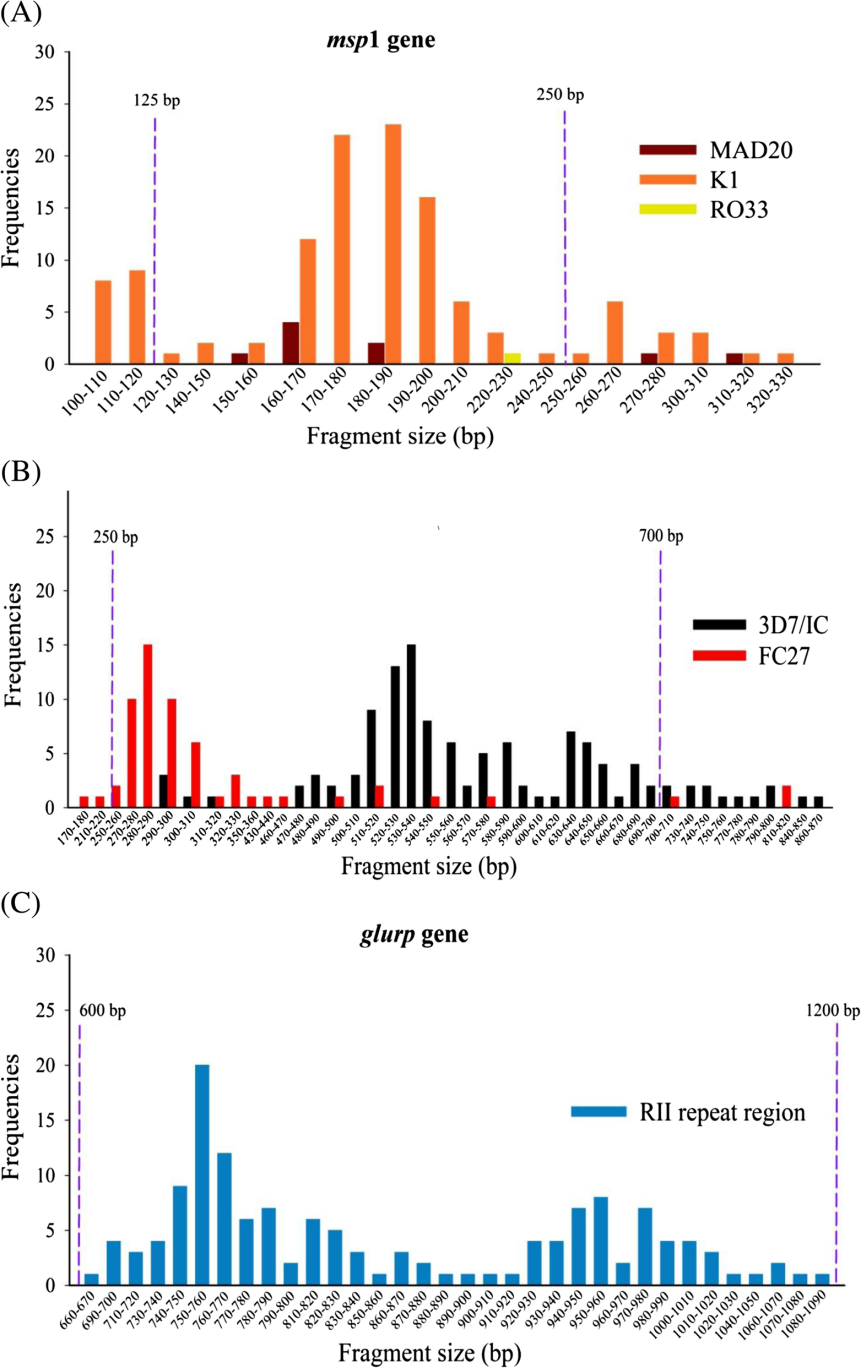
**Parasite genetic diversity detected by *msp1*, *msp2 *and *glurp*.** Polymerase chain reaction (PCR) products were categorized into molecular weight groups differing by 10 bp. **(A)***msp1*. Orange bars represent K1; brown bars, MAD-20; and yellow bars, RO33 allelic families. The 18 K1 alleles ranged in size from 100 to 330 bp; the 5 MAD-20 alleles; 150 to 320 bp; and the 1 RO33 alleles, 220-230 bp. **(B)***msp2*. Red bars represent FC27 and black bars represent IC3D7 allelic families. The 18 FC27 alleles ranged in size from 170 to 820 bp, and the 33 IC3D7 alleles ranged in size from 290 to 870 bp. **(C)***glurp*. The *glurp* alleles ranged in size from 660 to 1,090 bp. Dashed purple lines represent fragment size thresholds for each gene [10].

There was more genotypic variation in *msp2* (51 alleles) than *msp1* (24 alleles) and *glurp* (34 alleles) (Figure 1). The length variations of the *msp1*-amplified product were approximately 100-330 bp for K1, 150-320 bp for MAD20 and 220-230 bp for RO33. For *msp2*, the length variations of the amplified product were approximately 170-820 bp for FC27 and 290-870 for IC3D7, while *glurp* showed 660-1,090 bp of length variation.

### Haplotypes

**Figure 2:**
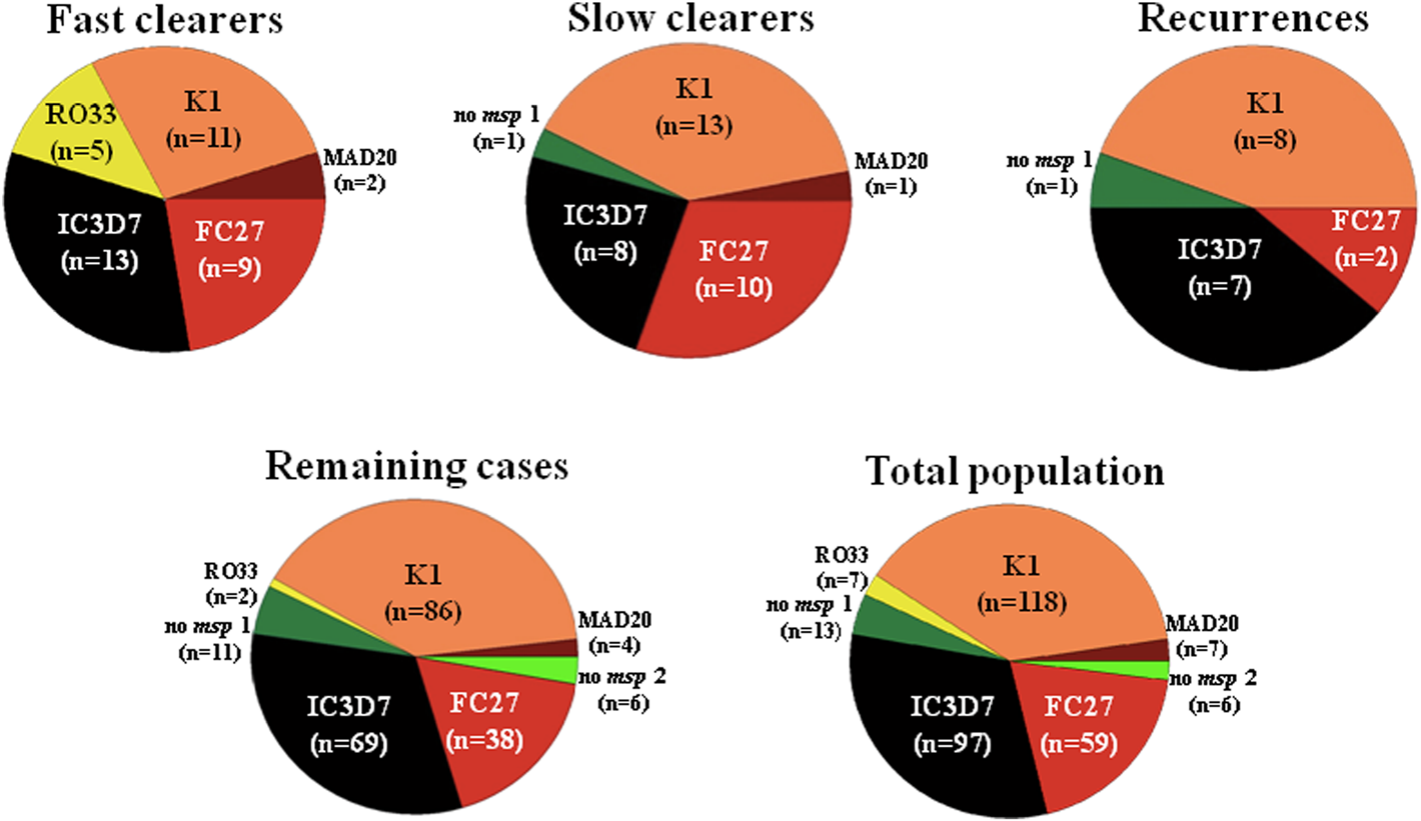
**Pie charts showing genotype frequencies of *msp1 *and *msp2 *determined at baseline for fast parasite clearers (artemisinin sensitive) (n = 14), slow parasite clearers (n = 18), recurrences (n = 9), and for remaining (n = 102) and overall (n = 143) study populations.** Key: orange, brown, yellow = *msp1*; dark green = no *msp1*; black, red = *msp2*; lime green = no *msp2.*

Some 13 distinct patterns of genotype (haplotypes) were observed at baseline (Tables 2 and 3, Figure 2). Recurrent cases were comprised of just three haplotypes: *msp1*-K1/*msp2*-IC3D7, *msp1-*K1/*msp2-*FC27 and *msp1-*MAD20/*msp2-*FC27; these three haplotypes were also prominent in Groups A and B, and therefore probably not associated with clinical phenotype. In contrast, haplotypes containing *msp2*-RO33 were observed in some AS-sensitive cases (5/18, 28%), but not in the AS-resistant subgroup.

**Table 2:** **Haplotypes of *msp1 *** **and *msp2 *** **seen in 143 cases of uncomplicated *P. falciparum *** **malaria**

**Type**	**Haplotype pattern**		**Polyclonal for >1 allele**	**Number of cases**				
	***msp1***	***msp2***		**Fast clearers**	**Slow clearers**	**Malaria recurrences**	**Remaining cases**	**Total population**
				**(n = 18)**	**(n = 14)**	**(n = 9)**	**(n = 102)**	**(n = 143)**
1	MAD20	IC3D7	No	1	-	-	1	2
			Yes	-	-	-	1	1
2	MAD20	FC27	No	-	-	-	1	1
			Yes	-	-	-	-	-
3	MAD20	IC3D7/FC27	No	-	-	-	-	-
			Yes	1	-	-	-	1
4	MAD20/K1	IC3D7/FC27	No	-	-	-	1	1
			Yes	-	1	-	-	1
5	K1	IC3D7	No	3	4	-	35	42
			Yes	3	**-**	7	13	23
6	K1	FC27	No	3	5	1	17	26
			Yes	-	-	-	6	6
7	K1	IC3D7/FC27	No	1	1	-	3	5
			Yes	1	2	-	5	8
8	K1	-	No	-	-	-	4	4
			Yes	-	-	-	2	2
9	RO33	IC3D7	No	2	-	-	-	2
			Yes	-	-	-	-	-
10	RO33	FC27	No	2	-	-	-	2
			Yes	-	-	-	-	-
11	RO33	IC3D7/FC27	No	1	-	-	1	2
			Yes	-	-	-	1	1
12	-	IC3D7	No	-	-	-	6	6
			Yes	-	-	-	2	2
13	-	FC27	No	-	1	1	3	5
			Yes	-	-	-	-	-

**Table 3:** **Number of patients with *msp1 *** **or *msp2 *** **genes detected at baseline**

** Genotype**	**Artemisinin sensitive**	**Slow parasite clearers**	**Recurrences**	**Remaining cases**	**Total population**
	**n = 18**	**n = 14**	**n = 9**	**n = 102**	**n = 143**
*msp1*					
MAD20	2 (29)	1 (14)	0	4 (57)	7
K1	11 (9)	13 (11)	8 (7)	86 (73)	118
RO33	5 (72)	0	0	2 (29)	7
No *msp1* detected	0	1 (8)	1 (8)	11 (85)	13
*msp2*					
IC3D7	13 (14)	8 (8)	7 (7)	69 (71)	97
FC27	9 (15)	10 (17)	2 (3)	38 (65)	59
No *msp2* detected	0	0	0	6 (100)	6

### Complexity of infection

**Figure 3:**
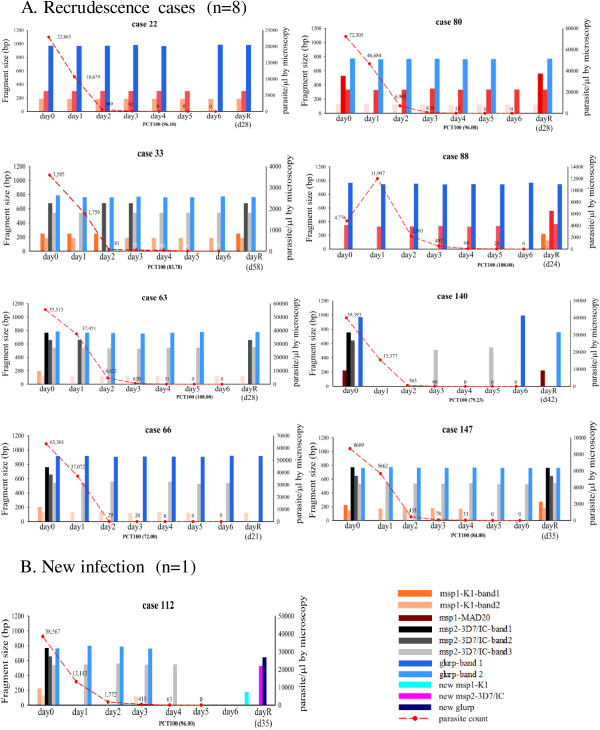
**Parasite genotype determined daily in individual patients with malaria recurrence (Group C).** Allele typing displaying size variation of 18 s alleles for *msp1, msp2* and *glurp* in *P. falciparum* parasites isolated from nine patients showing the patterns of alleles present during seven days of AS monotherapy and on the day of falciparum malaria recurrence. Key: Bp = base pairs; PCT100 = duration of parasite clearance by microscopy; bands = separate clones for the same allele; dayR = day of malaria recurrence. Dashed lines represent asexual parasitaemia determined by microscopy. **(A)** Recrudescence cases. Case 66 received only three days of initial AS treatment due to development of neutropenia and completed anti-malarial therapy with an alternative short-acting drug. All remaining cases completed seven days of AS monotherapy. Gametocytes were seen by microscopy for cases 33 (Day 0-21), 80 (Days 35 and 42), and 147 (Days 21 and 42). Case 88 had two shared alleles on D_f_ plus additional *msp1* and *msp2* loci. Case 140 had one new allele of *msp2* detected on Days 3 and 5 but not on Day 0 or D_f_. One shared *msp1* allele detected at D_f_ plus 1 new allele of *glurp.***(B)** New infection. Case 112 new alleles of *msp1, msp2* and *glurp* detected on D_f_ in addition to *Plasmodium vivax*.

Overall, 45/143 (31%) of the study population had evidence of a polyclonal infection at baseline with two or more *msp1, msp2 or glurp* alleles present (Tables 2 and 3). Multiple alleles were detected in 17% (21/125) *msp1*, 26% (36/138) *msp2* and 8% (12/143) *glurp* samples, respectively. The maximum number of clones seen for a single gene was six (one case with six clear bands of *msp2*-FC27 in addition to two clones of *msp1*-K1); this case remained cured at 42 days but had a prolonged parasite clearance time of 102 hours. All other polyclonal infections had just two or three clones. In the three subgroups of artemisinin sensitive, slow clearers and recurrences, multiple *P. falciparum* alleles of *msp-1, msp-2* and *glurp*, were detected in 5/18, 3/14 and 7/9 patients (28, 21 and 79%, respectively) (Figure 3). Recurrence patients were significantly more likely to have polyclonality of one or more alleles demonstrated at baseline than patients without falciparum recurrence (seven of nine *versus* 36 of 127, p = 0.004). Five of nine cases were polyclonal at D_f_.

### Parasite genotype during seven-day artesunate therapy and at failure

Parasite genotype was evaluated daily on Days 0-6 of AS therapy for individual recurrence cases (Figure 3). In all participants, peripheral parasitaemia cleared by Day 4 of AS therapy although genotyping PCR assay performed retrospectively demonstrated the continued presence of parasites through Day 6.

### Detection of minority parasite populations during treatment

Only one case of recurrence had a new allele (*msp2*-3D7) observed on Days 3 and 5, when asexual parasitaemia was still positive by light microscopy; this could represent a minority clone potentially relatively resistant to AS treatment. However, this allele was not detected again at D_f._ In the remaining eight recurrence cases, while fewer of the alleles present at baseline were detected as parasitaemia cleared, no new alleles emerged.

In the 14 patients with prolonged parasite clearance but no recurrence of malaria (Group B) no minority alleles emerged during seven days of treatment.

### Determination of parasitological outcome in recurrent malaria (LTF) cases

Based on conventional genotyping methodology using only baseline and D_f_ samples, eight of nine (89%) *Plasmodium falciparum* recurrences were determined to be recrudescent and one of nine (11%) was a new infection. Longitudinal genotyping on days 1-6 revealed that in six of nine cases no new minority parasite populations were detected during treatment, thus supporting the classification of recrudescence, although not all alleles present at baseline were detected at the time of recurrence. In one case (participant 140) described above, minority alleles were observed on Days 3 and 5 of AS treatment but not on D_f_. and thus the classification remained as recrudescence. One case (88) was also classified as recrudescent based on the appearance of at least one allele present in the baseline sample, as well as novel alleles at D_f_. Case 112 was classified as a new infection based on the appearance of novel alleles at the time of recurrence, none of which were observed during the first week. Thus in all nine cases of malaria recurrence, additional genotyping during AS treatment on days 1-6 did not alter classification of the final parasitological outcome.

## Discussion

This study aimed to detect emerging drug-resistant minority parasite clones during slowly clearing malaria infections that could account for cases of unrecognized treatment failure in a setting of clinical AS resistance. In fact, of the 23 cases in the artemisinin-resistant subgroup in whom daily parasite genotyping was undertaken for seven days, including nine cases with recurrent disease, a minority clone was detected in only one; moreover this clone was not present at D_f_, so did not appear to be associated with subsequent treatment failure. Two of the 23 cases had novel alleles present at D_f_ but these had not been detected at all during AS therapy despite the sensitivity of the molecular methods used. In this Cambodian setting, where most enrolled patients had persistence of asexual parasitaemia beyond 72 hours, the parasites responsible for slow parasite clearance and recurrent malaria are the dominant or co-dominant clones in an acute infection. Further, because they are also present in fast parasite clearers, they are well established in the Tasanh parasite population. These data also support recent reports of highly conserved, yet genotypically very distinct, populations of parasites from a number of sites in Cambodia including this study site [4].

In this setting of low malaria transmission and emerging drug resistance, considerable heterogeneity within the *P. falciparum* parasites was revealed in baseline samples from the symptomatic patient population. In keeping with surveys from West Africa and Thailand, these Cambodian isolates showed greater polymorphism of the *msp*2 allelic family (IC3D7 and FC27) compared to *msp*1 and *glurp*[12,16]. These results showing the distribution and diversity of *msp1, msp2* and *glurp* in a relatively isolated area of Western Cambodia are similar to those from directly across the border in Trat Province, Thailand [12]. Comparative data from other locations in Cambodia is currently lacking.

Evidence of polyclonal infection, in the form of the presence of two or more alleles from *msp1, msp2* and *glurp* within a single sample, was found in just 31% of subjects overall at baseline (17, 26 and 8% for *msp1, msp2* and *glurp*, respectively). This suggests a relatively low diversity of the *P. falciparum* parasite population in this localized area of Western Cambodia. A study from Kampala, Uganda, also a relatively low transmission area, found 45/98 (46%) samples were polyclonal for *msp2* at baseline [17], while in Kenya 60% of asymptomatic blood smear positive children in an area of moderate transmission in Kenya had two or more *msp2* clones detected [18]. For *msp1* 32% of 45 symptomatic and 64% of 45 asymptomatic infections in a hyperendemic region of Gabon had polyclonality of *msp1*[19]. An intriguing finding in this Cambodian study was that patients who subsequently developed recurrent disease were significantly more likely to harbour two or more clones at baseline compared with the rest of the study population. Since this is a location in which slow parasite clearance during AS treatment is the norm rather than the exception, the majority of slow clearers also had monoclonal infections; this raises a question as to whether, in addition to simply defining resistance as a certain proportion of cases with prolonged parasite clearance time or half-life, we should be broadening current research objectives to include identification and follow-up of recurrent polyclonal infections so as to define their role as potential agents of emerging resistance.

In the present study, six clones were demonstrated in one baseline sample, although only two or three clones were detected in the remaining 44 patients with polyclonal infections. In general, studies from high transmission areas have shown that asymptomatic malaria patients harbour more parasite clones than those with symptomatic disease [20]. The same is true for asymptomatic versus severe malaria: in a post-mortem study from Malawi, tissue genotyping for sequestered parasites demonstrated that children with cerebral malaria had sequestered parasite populations less genetically complex than both their circulating parasites and the circulating parasites in an asymptomatic malaria group, despite the much higher parasite burden [21]. The more homogeneous distribution of genotypes in cerebral malaria than in asymptomatic cases suggested the emergence of a single or small number of more dominant parasite clones, although the authors did acknowledge that the sheer burden of parasites in cases of cerebral malaria may have prevented lesser populations from being detected by PCR. Likewise, in this present study, conducted in a localized low transmission setting in Western Cambodia, cases of symptomatic disease, including most cases with very prolonged parasite clearance, tended to be dominated by single parasite clones. However, while there appeared to be an association of RO33 with some AS sensitive cases, no haplotype could be identified to be indicative of a resistant phenotype. Newer techniques, such as the heteroduplex tracking assay [22,23], may yield greater discrimination of clonal populations and identify haplotypes other than those used in this present analysis.

Additional daily samples were genotyped during seven days of AS treatment (Days 0 to 6) as well as on the day of recurrent *P. falciparum* parasitaemia (D_f_), which occurred between 21 and 58 days after commencing AS. Genotyping patterns from seven of nine treatment failure cases showed multiple parasite alleles of *msp1* and/or *msp2* at baseline, although only a minority were then detected during the following days of AS treatment. This may be due to greater sensitivity of some parasite strains to AS treatment, as well as to declining parasitaemia in the small volume of sampled blood, or to a combination of both. An alternative explanation for the disappearance of some parasite strains, which later re-appear at the time of treatment failure, is parasite dormancy triggered by the presence of AS [24,25].

However, repeated genotyping of symptomatic travellers returning from Africa before and during 96 hours of quinine treatment for *P. falciparum* malaria in France revealed 19/20 (95%) cases harboured between two and five clones, with some cases having up to three dominant clones in addition to other minority clones [26]. Moreover, quantification of clones revealed that while the parasitaemia of some declined steadily during drug treatment, others showed major fluctuations of parasitaemia that were apparently unrelated to the presence of drug. Thus, a single baseline blood sample may provide incomplete information on the diversity of parasite populations present within a given patient. In contrast, Färnert and colleagues found only one of 13 patients with symptomatic malaria had new clones detected sporadically during 12-hourly sampling over a three to nine-day period, despite more than half (seven of 13) having multiple clones of *msp1* and *msp2* detected at baseline [27]. This latter study reflects the findings in this study in which just one of nine treatment failures and none of 14 slow parasite clearers had new alleles detected during treatment.

A striking feature in patients undergoing longitudinal sampling over seven days was the presence of parasite DNA up to seven days after the initiation of AS therapy; this may represent relatively drug-resistant *P. falciparum* populations undergoing selection or modification in the presence of drug. Parasite DNA is detectable in peripheral blood for less than 48 hours following the death of a parasite in response to drug or immune mechanisms and before being removed from the circulation by circulating phagocytes and spleen macrophages [28]. Therefore the presence of parasite DNA in blood beyond the microscopy-calculated parasite clearance time does likely represent the persistence of viable parasites. As well as the increased sensitivity of PCR compared to microscopy (in general 0.1 *versus* 10 parasites/μL [29]), an additional explanation for the persistently positive PCR results up to the final day of AS treatment is the presence of gametocytes in the blood samples. However, in this study only one of 23 patients in the AS-resistant group had gametocytes detected by microscopy during the first seven days, and just one of nine LTF cases had gametocytes on D_f_. Therefore the quantification results are unlikely to be influenced significantly by the presence of circulating gametocytes. Using current PCR strategies, gametocytes can only be detected differentially by reverse transcription-PCR (RT-PCR) [30]. The ability to differentiate gametocytes from asexual parasites would enable a more reliable estimate of true failure rates and thus support efforts to limit the spread of drug resistance and meet containment and elimination targets.

In this study, conventional genotyping assigned the outcome of six of nine samples as recrudescence; additional genotyping on Days 1-6 did not alter this assessment. In two further cases, while the final classification was not altered by additional genotyping the presence of additional bands does illustrate some of the dilemmas inherent in this genotyping technique. Mixed genotype results may profoundly impact estimates of drug resistance if they form a significant proportion of treatment failure cases in clinical trials, and depending on how they are interpreted [8]. If circulating strains at low parasite densities are not detected in baseline samples because other (dominant) parasite strain(s) are present in much larger quantities, they could still emerge later to cause treatment failure, leading to the misclassification of a recrudescence as a new infection and underestimating the amount of drug resistance in the patient population [30]. The argument for analysing genotypes from Day 1 as well as Day 0 samples for comparison with D_f_ has been made previously and may be particularly valuable in high transmission settings [17,31]. Conversely, due to incomplete immunity resulting from a single treated malaria infection, patients could naturally re-acquire malaria caused by the same parasite genotype if they return to an endemic area with relatively little parasite genetic diversity, this time leading to an over-estimate of drug resistance. Current classification schemes of alleles for use in assigning treatment outcome are limited and often subjective and varying between observers. A recent MMV/WHO consensus report on *P. falciparum* genotyping recommends possible use of polyacrylamide gels, capillary electrophoresis, or use of dedicated fragment sizing software (used here), to increase discriminatory power and reduce inconsistencies between clinical trial methodologies [8].

There are several limitations to this analysis. More cases of treatment failure, in particular of new infections with *P. falciparum* would have enabled potential misclassification of outcome to be examined in more detail. Additional emergent minority populations may have been detected if sampling had occurred more frequently than once daily. Band size estimates, while measured by automated reader with as much precision as possible, may also have led to minor inaccuracies. Moreover band size variability between the same alleles can potentially occur, leading to false categorization as different alleles. Analysis of samples in this study was limited to once per day so minority parasite clones with only a sporadic and low-level presence in the peripheral circulation compared to the dominant clone(s) could have been missed.

## Conclusion

Limited genotyping of *msp1, msp2* and *glurp* in malaria patients from a localized area of Western Cambodia revealed considerable variation in *msp2* alleles but well-conserved *msp1* and *glurp*. Longitudinal genotyping of cases with recurrent malaria during seven days of anti-malarial treatment showed homogeneous genotype dynamics and did not alter the outcome classification compared to conventional genotyping at baseline and D_f_; however it did serve to illustrate some of the inherent complexities when interpreting the parasitological basis of recurrent disease. In contrast to Africa, where malaria transmission rates are higher and artemisinin resistance does not currently appear problematic, lower polyclonality of infection was observed in malaria patients in Cambodia. The exception was the group of recurrent malaria infections in which polyclonality was much higher. The potential role of polyclonal infections in contributing to the spread of artemisinin resistance deserves further investigation.

## Competing interests

The authors declare that they have no competing interests.

## Authors’ contributions

DB conceived the study; PG, SS, SW, KP, and ST performed the molecular genetic studies; YS, CL, SS, SD, MF, DS, DB conducted the clinical trial; PG, CL and DB analysed the data; PG, CL, ST, MS, CMC, DW, MF, JM, DS, and DB wrote the manuscript. All authors read and approved the final manuscript.
